# The Clinical Outcome of Cardiac Rehabilitation in Coronary Artery Disease Patients with Regard to the Presence of Left Ventricular Systolic Dysfunction

**DOI:** 10.3390/jcm13102969

**Published:** 2024-05-17

**Authors:** Iwona Szadkowska, Katarzyna Szmigielska

**Affiliations:** 1 Department of Sports Medicine, Medical University of Lodz, 92-231 Lodz, Poland; iwona.szadkowska@umed.lodz.pl; 2 Outpatient Rehabilitation Unit, Central Teaching Hospital of the Medical University of Lodz, Pomorska 251, 92-231 Lodz, Poland

**Keywords:** coronary artery disease, heart failure, ejection fraction, cardiac rehabilitation, exercise

## Abstract

**Background**: Despite the use of advanced treatment techniques, coronary artery disease (CAD) still remains the main cause of left ventricular (LV) dysfunction and heart failure. Participation in cardiac rehabilitation (CR) programs can lead to a number of beneficial effects, but some patients do not demonstrate the expected improvement. The aim of this study is to evaluate the impact of CR on changes in exercise capacity with regard to the presence of LV dysfunction. **Methods**: A group of 428 patients with CAD were consecutively admitted to an outpatient comprehensive cardiac rehabilitation program comprising 24 exercise sessions of interval training on cycle ergometers, three times a week for 45 min, and a health education. The patients were compared in two subgroups, i.e., with LV systolic dysfunction (LVEF < 50%, *n* = 175) and LVEF ≥ 50% (*n* = 253). **Results**: In the LVEF < 50% group, the exercise capacity improved by 1 ± 0.78 MET (median 1.15 MET), and 0.86 ± 0.77 MET (median 1.08 MET) in the LVEF ≥ 50% group. Women with LVEF < 50% demonstrated a significant increase in exercise capacity by 1.2 MET, while those with LVEF ≥ 50% did not display any such increase. All men, regardless of LVEF, exhibited a similar improvement in exercise capacity greater than 1 MET. **Conclusions**: An outpatient eight-week cardiac rehabilitation program based on 45 min aerobic interval training sessions three times a week appears less effective for women with CAD and EF ≥ 50%. In this group, the proposed training intervention is insufficient in improving exercise capacity to an extent that could indicate a reduction in mortality risk.

## 1. Introduction

Cardiovascular diseases are widespread and still represent the dominant cause of death in developed countries [1]. Coronary artery disease (CAD), particularly myocardial infarction, can lead to systolic dysfunction of the left ventricle (LV), resulting in the development of heart failure (HF)—with a reduced or mildly reduced left ventricular ejection fraction (LVEF). Moreover, CAD is one of the most common causes of HF with preserved LVEF (HFpEF) [2,3,4].

Currently, the incidence of HF is increasing. This has been attributed to the rising prevalence of cardiometabolic disorders, obesity, and a sedentary lifestyle [1,2,3,5,6]. Non-pharmacological management, including regular physical activity, can play a major role in the primary and secondary prevention of both CAD and HF [7]. Moreover, modern invasive methods used in the treatment of CAD are aimed at preventing the development of myocardial damage, and improving the early and long-term prognosis [8]. However, there is still a problem in clinical practice where these large-scale management strategies aimed at disease prevention and diagnosis, and new therapeutic methods, have limited effectiveness in some individuals.

A comprehensive approach to this problem is offered by cardiac rehabilitation (CR) programs based on regular physical activity, which can lead to number of beneficial changes, including an improved blood lipid profile, reduced blood pressure and body weight, and increased exercise capacity and VO2max. However, despite the implementation of comprehensive management, not all patients fully benefit from CR, which can be associated with various factors [3,9,10,11,12,13].

Studies have confirmed that the presence of LV dysfunction is associated with poorer overall clinical condition and worse prognosis [1,2,3,4,14,15,16]. On the other hand, the exercise capacity of patients with CAD or HF is also of great importance, as an indicator of general clinical status and prognosis [3,4,9,10,11,12,13].

Therefore, the aim of this study is to evaluate the effect of cardiac rehabilitation on the exercise tolerance parameters of patients with CAD, with regard to the presence of LV dysfunction.

## 2. Materials and Methods

This study included 428 patients, 296 males and 132 females, aged 31 to 87 years (mean age 62.6 ± 9.6). The participants were consecutively admitted to an outpatient comprehensive cardiac rehabilitation program after a cardiac event—acute coronary syndrome (ACS), percutaneous coronary interventions (PCI), or coronary artery bypass surgery (CABG). All participants were treated according to current recommendations depending on their clinical status.

In order to determine the effects of CR depending on the presence of LV dysfunction, patients were compared in subgroups: with LVEF < 50% and LVEF ≥ 50% [3,4].

Only data of patients who completed the entire eight-week comprehensive CR program were used in the statistical analysis.

### 2.1. Comprehensive Cardiac Rehabilitation Program

The comprehensive cardiac rehabilitation (CR) program was based on exercise sessions and an educational element aimed at informing the participants about cardiovascular risk factors, lifestyle modifications such as a healthy diet and physical activity, and psychological support.

The exercise sessions consisted of 24 interval trainings on cycle ergometers (Ergoline Reha System GmbH, Schiller, Switzerland) three times a week for 45 min. Each training session was made up of four-minute workloads separated by two minutes of active restitution, with the workload increasing during the first part and decreasing during the second. During the training sessions, blood pressure and electrocardiographic values were continuously monitored.

For each patient, the training heart rate (THR) was calculated according to the heart rate reserve (HRR) as follows:

THR = (0.5 to 0.7) × HRR + resting HR

HRR = the highest HR achieved during the exercise test − resting HR.

### 2.2. Data Collection

All participants, after a physical examination, underwent an exercise test, at the beginning of the CR program and after eight weeks. Body weight and height were measured using a WPT 100/200 digital scale (Radwag) and a stadiometer (GMP, Switzerland), based on standard anthropometric methods. Body mass index (BMI) was calculated according to the formula:

BMI = weight/height^2^ (kg/m^2^).

The multistage, symptom-limited exercise tests were performed on an Ergoselect II 100/200 cycle ergometer with continuous 12-lead electrocardiographic monitoring using a Cardiovit CS-200 Ergo Spiro (Schiller, Switzerland). At the beginning of the exercise test, the workload was set at 60 Wat and was gradually increased by 30 Wat every three minutes until exhaustion. When the exercise phase was completed, the patients were monitored for five minutes or longer, during a cooldown phase, which consisted of two minutes of pedaling without workload and then three to five minutes of rest.

The exercise tests was terminated upon exhaustion or the occurrence of any of the following clinical symptoms: chest pain, dizziness or headache, breathing difficulties, >250 mmHg systolic BP or >115 mmHg diastolic BP, or abnormal ECG findings.

Blood pressure was measured at rest and at the end of each stage of the exercise test. The peak heart rate (HRpeak) was defined as the highest heart rate reached during the exercise test. The rate pressure product (RPP) was calculated as HR × systolic BP. The highest values measured during the exercise test were used to calculate RPPpeak, while the resting RPP was based on resting values. Exercise capacity was defined as the highest MET value obtained during the exercise test. The highest workload performed during the exercise test, expressed in Wats and Wats/kg, was considered peak workload (Wpeak). Also, in the last stage of the exercise test, perceived exertion was assessed according to the 20-point Borg scale.

### 2.3. Echocardiography

Transthoracic echocardiography was performed in all patients using a Vivid S70 ultrasound system (General Electric Healthcare, Chicago, IL, USA, 2018) after the end of the eight-week rehabilitation program. The cardiac parameters were assessed according to the current recommendations [17]. The findings were compared with the echocardiographic data obtained during hospitalization which was an indication for CR.

### 2.4. Statistical Analysis

Continuous variables are presented as mean ± standard deviation or as median with interquartile range, as appropriate. The initial and final values in each group were compared using a paired *t*-test for parametric data, or Wilcoxon’s test for nonparametric data.

When analyzing differences in continuous variables between groups, a two-sample Student’s *t*-test was used for variables with a normal distribution, and the Mann–Whitney U-test for those without a normal distribution. Statistical significance was defined as a *p*-value below 0.05. The statistical analysis was performed with Statistica software, Version 13.1, USA.

## 3. Results

The study population consisted of 428 patients with CAD, consecutively enrolled to an outpatient CR program (with a mean time 23.2 ± 9.0 days after hospitalization). Patients were divided into two groups based on the presence of LV dysfunction: 175 patients with LV dysfunction (LVEF < 50%) and 253 with preserved ejection fraction (LVEF ≥ 50%). All subjects enrolled into this study were treated according to current recommendations depending on the clinical status.

The participants did not differ significantly, taking into account the age, cardiovascular risk factors, and clinical history including the comorbidities. The percentage of women in both groups was similar. However, hypertension was more common in the group with preserved LVEF. The justification for inclusion in CR was most commonly No ST Elevation Myocardial Infarction (NSTEMI) in the LVEF ≥ 50% group, and ST Elevation Myocardial Infarction (STEMI) in the LVEF < 50% group; these differences were statistically significant (Table 1).

The body mass index significantly increased over the program in the preserved LVEF group, while no change in BMI was found in the LVEF < 50% group. Waist circumference did not change in either group over the CR program. Hemodynamic parameters measured at rest, viz., systolic and diastolic blood pressure and heart rate, decreased significantly after CR as compared to baseline in both groups. Peak workload during the exercise test (in Wat and in Wat/kg) as well as exercise capacity expressed in METs increased significantly after eight weeks of CR in both groups.

The mean improvement in exercise capacity was 1 ± 0.78 MET (median 1.15 MET) in the LVEF < 50% group, and 0.86 ± 0.77 MET (median 1.08 MET) in the LVEF ≥ 50% group; however, these differences were not statistically significant (Table 2).

To understand why lower benefits may be achieved from cardiac rehabilitation, the participants were divided into two subgroups depending on the degree of increase in exercise capacity. The first group consisted of patients whose increase in exercise capacity was lower than 1 MET (*n* = 160), and the second group consisted of those whose increase in exercise capacity was greater than 1 MET (*n* = 268). It was found that the group of patients achieving an increase in exercise capacity of less than 1 MET had a significantly higher BMI compared to the other group. The comparison of these two subgroups is presented in Table 3.

**Figure 1:**
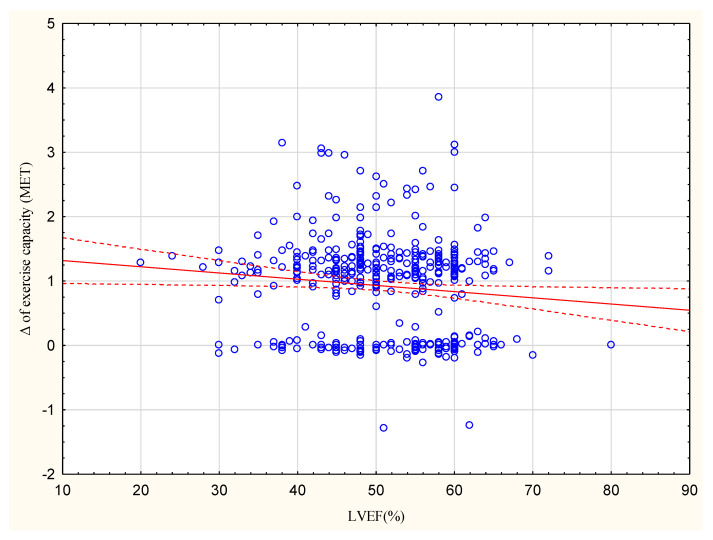
Correlation between the increase in exercise capacity in METs and LVEF (%); R = −0.1078.

The increase in exercise capacity in METs significantly correlated with BMI at R = −0.1385, *p* < 0.05 (Figure 1).

**Figure 2:**
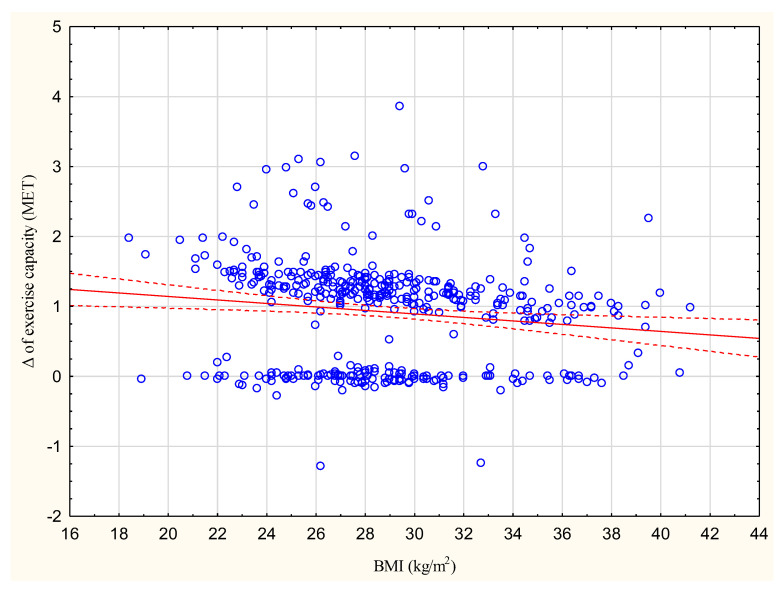
Correlation between the increase in exercise capacity in METs and BMI (kg/m^2^); R = −0.1385.

The group with an increase <1 MET had a higher proportion of patients with LVEF ≥ 50% (64%) compared to the group with an increase >1 MET (44%; Table 3). The observed increase in exercise capacity in METs correlated with LVEF at R = −0.1078, *p* < 0.05 (Figure 2). 

The group with an increase <1 MET included a significantly higher proportion of women compared to the other group (Table 3). The women who were more often examined presented an increase in exercise capacity lower than 1 MET in comparison to examined men. Among the women, only those with LVEF < 50% demonstrated a significant increase in exercise capacity, i.e., by 1.2 MET. In contrast, those with LVEF ≥ 50% experienced an increase of 0.0 MET. The men demonstrated similar improvements in exercise capacity regardless of LVEF: a 1.15 MET increase for LVEF < 50% and a 1.2 MET increase for LVEF ≥ 50% (Table 4).

## 4. Discussion

Cardiac rehabilitation (CR) is an essential stage in the comprehensive management of patients with various cardiovascular diseases. When applied as a personalized and supervised physical training program, together with parallel interventions provided by a multidisciplinary team, it is known to achieve particularly beneficial effects. Participation in a CR program has been found to result primarily in a reduced risk of mortality; this is, above all, associated with the improvement in physical performance, correction of risk factors, and optimization of pharmacotherapy [3,7,8,18,19,20].

However, it has also been observed that not all patients respond to CR to the same extent, especially among HF patients. It has been proposed that this may be due to the fact that the population participating in CR demonstrates high heterogeneity in relation to age, sex, degree of cardiac damage, or concomitant diseases. Hence, it is extremely important to identify such patients who may have an increased probability of inadequate response to CR [21,22,23,24,25].

The presence of LV dysfunction is a key predictor of the effectiveness of treatment in patients with cardiovascular diseases. Individuals with reduced LVEF are characterized by a higher mortality rate, as well as poorer exercise capacity and quality of life. In CR models, LVEF is a basic element of a patient’s risk assessment [3,8,14,18,21].

In the present study, 41% of patients with CAD undergoing invasive treatment procedures were characterized by LV dysfunction. While observational studies have found that a minority of all patients with CAD demonstrate LVEF < 50%, the exact value may vary depending on the population analyzed [14,26,27]. In the present study, individuals with preserved LVEF were more likely to have hypertension and previous NSTEMI; however, no significant differences regarding sex or other clinical features were found between groups with and without LV dysfunction.

Regardless of baseline LVEF value, the CR program yielded significant improvements in resting and exercise hemodynamic parameters, and improved exercise tolerance and LVEF values, which indicates its beneficial effects. However, only an improvement in exercise capacity above 1 MET is considered clinically significant, and the mean values do not identify a subgroup with an unsatisfactory intervention effect.

Exercise capacity has been shown to be a stronger predictor of mortality risk than traditional factors such as hyperlipidemia, hypertension, type 2 diabetes, obesity, and smoking. It is also superior to other exercise test parameters, like exercise-related symptoms, ST segment depression, and hemodynamic responses to exercise [28,29].

It has been assumed that an increase in exercise capacity by 1 MET reflects a decrease in all-cause mortality by between 12% and 26% [11,12,30].

To identify the factors reducing the effectiveness of CR, two subgroups of patients were created depending on the achieved increase in exercise capacity. Of the 428 participants, 160 (37%) achieved less than a 1 MET increase. This group was characterized by a higher BMI value, higher percentage of patients with LVEF ≥ 50%, and higher percentage of women compared to the group achieving more than 1 MET.

In addition, a higher BMI was found to be connected with a lower increase in exercise capacity, although this relationship was not strong; however, previous studies do not indicate that BMI may have an influence on the increase in exercise capacity after CR [31,32]. In the present study, neither BMI nor waist circumference improved significantly after eight weeks of CR in any of the analyzed subgroups, as also noted previously [31,32].

Current recommendations indicate that in order to reduce body weight, aerobic physical activity should be undertaken for more than 150 min a week [10]. Moreover, it is possible that the current program, i.e., eight weeks of aerobic training performed three hours a week, is insufficient in inducing effective weight loss.

In the present study, the group achieving <1 MET included a higher percentage of patients with LVEF ≥ 50% (64%) than the >1 MET group (44%). Moreover, a higher LVEF correlated with a lower increase in exercise capacity, although this relationship was not strong (R = −0.1078).

Previous studies indicate a relationship between left ventricular dysfunction and increased exercise capacity resulting from a CR program [33,34]. In addition, exercise-based rehabilitation has been found to yield an improved exercise capacity in patients with heart failure with a reduced ejection fraction, as noted in meta-analyses and clinical trials [35,36], with the most significant increase in exercise capacity being observed in patients with the lowest exercise capacity (peakVO2 < 20 mL/kg/min) [34].

In the present study, the <1 MET group included a significantly higher proportion of women compared to the >1 MET group. In addition, the women were more likely than men to achieve an increase in exercise capacity <1 MET. Interestingly, previous studies report significant increases in exercise capacity after CR in both sexes [37,38]; however, others found a higher improvement in exercise capacity after CR in men than women [39,40,41]. Many studies have found that women are less likely to be referred and to participate in CR for various reasons [10,39,42,43,44,45]. In our investigation also, significantly fewer women than men participated in the CR program.

It has been found that women more often have a worse risk factor profile than men [39,44,45,46] and are less likely to receive preventive treatment than men with a similar risk of atherosclerosis [39,47]. Also, women were less likely to achieve optimal results in the treatment of lipid disorders or hypertension, and pharmacotherapy was often found to be less aggressive [42,48,49].

In the present study, women with LVEF < 50% demonstrated a significant increase in exercise capacity, i.e., by 1.2 MET, while those with LVEF ≥ 50% did not exhibit any such increase. The men, however, achieved similar improvements in exercise capacity (>1 MET) regardless of LVEF.

The differences in outcomes of CR between women and men with CAD might be because the origin of myocardial ischemia differs by gender [50,51]. Men and women are characterized by different degrees of CAD morbidity and mortality. Female ACS patients typically have more comorbidities, are older, and receive suboptimal treatment compared to their male counterparts [39,42,50,51]. Coronary atherosclerosis in women may also be more diffuse. Furthermore, in women, ischemic heart disease may not be only atherosclerotic obstructive CAD, but can also be associated with vasomotor abnormalities, endothelial dysfunction, or coronary microcirculation dysfunction [50,51].

The answer to this problem may be the use of new echocardiography techniques and more sensitive parameters of myocardial dysfunction in further research, especially in the individuals with preserved LVEF. Studies in recent years have shown that both global longitudinal strain and myocardial work can be early indicators of improvement in myocardial function after a cycle of physical training, independently of other clinical factors [52,53]. Their analysis in the context of physical performance could provide additional information influencing the overall management and prognosis assessment, especially in the group of patients who do not achieve significant clinical improvement after CR.

The main findings of our investigation confirm that the participation of women in CR remains insufficient, and that the results achieved in this group vary considerably [24]. Even so, our data show that women with preserved EF tend to achieve suboptimal results for typical CR programs.

Extending the duration or volume of the supervised training program and strengthening the role of education in maintaining the level of physical activity in the long term are both important goals, as these will not only lead to an improvement in exercise capacity, but also reduce body fat mass. This is of great prognostic importance, as BMI and the level of physical activity are the main factors associated with the risk of HFpEF development [54].

## 5. Limitations

The lack of a matched control group is the limitation of our study. Creating a control group was not possible, because it would be unethical to deny cardiac rehabilitation to any patient with any indication, and those who refuse to participate in the CR program are not willing to take part in control visits. We also realize that our study is solely observational.

## 6. Conclusions

An outpatient eight-week CR program based on 45 min aerobic interval training sessions three times a week appears less effective for women with CAD and preserved LVEF. In this group, the proposed training intervention is insufficient in improving exercise capacity to an extent that could indicate a reduction in mortality risk.

## Figures and Tables

**Table 1:** General baseline characteristics of studied population in groups with LVEF < 50% and LVEF ≥ 50%.

Study Participants (*n* = 428)	Patients with LVEF < 50% (*n* = 175)	Patients with LVEF ≥ 50% (*n* = 253)	*p*
Age (years)	63.02 ± 9.04 64 (34–87)	62.38 ± 10.04 64 (31–85)	0.8
Women, *n* (%)	46 (26)	86 (34)	0.08
Men, *n* (%)	129 (74)	167 (66)	0.08
BMI (kg/m^2^)	28.4 (18.4–40.8)	28.1 (19.1–41.2)	0.8
Waist circumference (cm)	102 (70–136)	100 (70–128)	0.1
Clinical history			
STEMI, *n* (%)	109 (62)	59 (23)	0.0000001
NSTEMI, *n* (%)	60 (34)	155 (61)	0.000001
PCI, *n* (%)	159 (90)	209 (83)	0.14
CABG, *n* (%)	29 (16)	47 (18)	0.57
Duration of CAD (years)	2.45 ± 5.55 0 (0–27)	2.57 ± 5.05 0 (0–24)	0.87
Single-vessel disease, *n* (%)	71 (40)	108 (43)	0.8
Two-vessel disease, *n* (%)	54 (30)	77 (30)	0.8
Three-vessel disease, *n* (%)	50 (28)	68 (23)	0.8
Diabetes mellitus, *n* (%)	46 (26)	69 (27)	0.8
Arterial hypertension, *n* (%)	132 (75)	223 (88)	0.0006
Smokers/ex-smokers, *n* (%)	0/90 (51)	0/119 (47)	0.3
LVEF baseline (%)	45 (20–49)	56 (50–80)	0.0000001

BMI—body mass index. CABG—coronary artery bypass grafting. CAD—coronary artery disease. LVEF—left ventricular ejection fraction. STEMI—ST Elevation Myocardial Infarction. NSTEMI—No ST Elevation Myocardial Infarction. PCI—percutaneous coronary intervention. Values are presented as median and (interquartile range)/a mean ± standard deviation.

**Table 2:** Anthropometric, hemodynamic, and exercise tolerance parameters of patients before and after CR in LVEF groups.

Study Population (428)	Patients with LVEF < 50% (*n* = 175)			Patients with LVEF ≥ 50% (*n* = 253)		
	Baseline	After CR	*p*	Baseline	After CR	*p*
BMI (kg/m^2^)	28.4	28.3	0.01	28.1	28.4	0.003
	(18.4–40.8)	(18.4–40.3)		(19.1–41.2)	(19.4–44)	
Waist circumference (cm)	102	102	>0.05	100	100	>0.05
	(70–136)	(70–136)		(70–128)	(68–144)	
HR at rest (beats × min^−1^)	71	70	0.004	72	70	0.0003
	(51–100)	(49–100)		(46–103)	(48–120)	
SBP at rest (mmHg)	120	117.5	0.000003	120 *	120 **	0.00001
	(195–160)	(90–160)		(90–150)	(90–160)	
DBP at rest (mmHg)	80	75	0.004	80	75	0.00002
	(60–90)	(50–90)		(60–100)	(50–90)	
Resting RPP × 10^−2^ (beats × min^−1^ × mmHg)	85.2	79.8	0.000001	84	84	0.000001
	(57–138.6)	(49.5–123.5)		(53–144)	(56.7–134.4)	
Peak workload (W)	90	120	0.0000001	120	120	0.000001
	(30–150)	(30–180)		(30–180)	(30–240)	
Peak workload (W/kg)	1.02	1.29	0.0000001	1.0	1.27 **	0.000001
	(0.39–2)	(0.39–2.5)		(0.33–2.14)	(0.39–2.86)	
Exercise capacity (MET)	4.42	5.44	0.0000001	4.45	5.35	0.000001
	(2.33–7.85)	(1–9.57)		(2.14–8.34)	(2.33–10.79)	
Δ of exercise capacity (MET) *	1 ± 0.78			0.86 ± 0.77		
	1.15 (−2.42–3.14)			1.08 (−1.29–3.8)		
RPP*peak* × 10^−2^ (beats × min^−1^ × mmHg)	170.8	180	0.008	174.4	189.35 **	0.000001
	(66–302.5)	(96.2–268.4)		(100.8–285.6)	(110.6–297)	
RPE (points)	14	15	>0.05	15	16	>0.05
	(13–17)	(13–17)		(13–18)	(14–17)	
LVEF (%)	45 (20–49)	48 (28–66)	0.00001	56 (50–80)	58 (43–85)	0.00001

BMI—body mass index. CR—cardiac rehabilitation. DBP—diastolic arterial blood pressure at rest (mmHg). HR—heart rate at rest (beats/min). LVEF—left ventricular ejection fraction. Peak workload—workload during the last stage of exercise test. RPE—rating of perceived exertion on 20-point Borg scale during the last stage of exercise test. RPP—rate pressure product (HR × SBP). RPP*peak*—rate pressure product (HR*peak* × SBP*peak*—the highest values achieved during the last stage of exercise test). SBP—systolic arterial blood pressure at rest (mmHg). * *p* < 0.05 group with LVEF < 50% versus group with LVEF ≥ 50% at baseline. ** *p* < 0.05 group with LVEF < 50% versus group with LVEF ≥ 50% after 8 weeks of CR. Values are presented as median and (interquartile range).

**Table 3:** Anthropometric parameters, risk factors, and clinical history of studied patients before CR in relation to changes in exercise capacity in MET.

Study Participants (*n* = 428)	Patients with Δ of Exercise Capacity < 1 MET (*n* = 160)	Patients with Δ of Exercise Capacity ≥ 1 MET (*n* = 268)	*p*
Age (years)	63.95 ± 8.59 65 (35–83)	61.85 ± 10.17 64 (31–87)	>0.05
Women, *n* (%)	72 (45)	60 (22)	0.0001
Men, *n* (%)	88 (55)	208 (77)	0.0001
BMI (kg/m^2^)	29.48 ± 4.5 28.95 (18.9–40.8)	28.44 ± 4.01 28.1 (18.4–41.2)	0.001
Waist circumference (cm)	101 (70–136)	101 (74–127)	0.1
Exercise capacity (MET)	4.4 (2.3–7.1)	4.5 (2.1–8.3)	>0.05
Δ of exercise capacity (MET)	0.1 ± 0.34 0 (−1.29–0.94)	1.4 ± 0.45 1.3 (1.0–3.9)	0.000001
LVEF (%)	52.02 ± 8.74 54 (30–80)	50.48 ± 8.4 50 (20–72)	0.057
LVEF < 50% (*n*; %)	57 (35)	150 (56)	0.0001
LVEF ≥ 50% (*n*; %)	103 (64)	118 (44)	
Clinical history			
STEMI, *n* (%)	64 (40)	104 (38)	>0.05
NSTEMI, *n* (%)	48 (30)	139 (52)	>0.05
PCI, *n* (%)	135 (84)	230 (86)	>0.05
CABG, *n* (%)	29 (18)	47 (17)	>0.05
Duration of CAD (years)	2.62 ± 5.36 0 (0–27)	2.44 ± 5.2 0 (0–27)	>0.05
Single-vessel disease, *n* (%)	67 (41)	112 (42)	>0.05
Two-vessel disease, *n* (%)	52 (32)	79 (29)	>0.05
Three-vessel disease, *n* (%)	41 (25)	76 (28)	>0.05
Diabetes mellitus, *n* (%)	43 (27)	71 (26)	>0.05
Arterial hypertension, *n* (%)	140 (87)	214 (79)	>0.05
Smokers/ex-smokers, *n* (%)	0/85 (53)	0/124 (46)	>0.05

BMI—body mass index. CABG—coronary artery bypass grafting. CAD—coronary artery disease. CR—cardiac rehabilitation. NSTEMI—No ST Elevation Myocardial Infarction. PCI—percutaneous coronary intervention. STEMI—ST Elevation Myocardial Infarction. Values are presented as median and (interquartile range) or a mean ± standard deviation.

**Table 4:** The comparison of patients’ exercise capacity at baseline and after CR according to sex and LVEF.

		LVEF < 50%	LVEF ≥ 50%	*p*
Women	Exercise capacity (MET)			
	- Baseline - After CR - Δ of exercise capacity	3.97 ± 0.73; 3.94 (2.33–6.18)	4.13 ± 0.97; 4.02 (3.14–6.61)	0.55
		4.91 ± 0.97; 4.92 (2.3–6.9)	4.6 ± 1.0; 4.42 (2.33–7.63)	0.06
		0.9 ± 0.85; 1.2 (−0.1–3.1)	0.5 ± 0.67; 0.0 (−1.2–2.0)	0.03
	*p* (women: baseline vs. after CR)	0.000001	0.000001	
Men	Exercise capacity (MET)			
	- Baseline - After CR - Δ of exercise capacity	4.58 ± 0.87; 4.62 (2.35–7.85) *	4.78 ± 0.9; 4.72 (2.19–8.34) *	0.12
		5.67 ± 1.07; 4.5 (3.18–9.5) *	5.8 ± 1.07; 5.72 (2.87–10.79) *	0.17
		1.0 ± 0.7; 1.15 (−0.15–3.05)	1.1 ± 0.75; 1.2 (−1.3–3.9) *	0.6
	*p* (men baseline vs. after CR)	0.000001	0.000001	

CR—cardiac rehabilitation. LVEF—left ventricular ejection fraction. * *p* < 0.05 women in comparison to men.

## Data Availability

Available on demand.
